# Stability of frontal alpha asymmetry in depressed patients during antidepressant treatment

**DOI:** 10.1016/j.nicl.2019.102056

**Published:** 2019-10-31

**Authors:** Nikita van der Vinne, Madelon A. Vollebregt, Michel J.A.M. van Putten, Martijn Arns

**Affiliations:** a Research Institute Brainclinics, Nijmegen, The Netherlands; b Synaeda Psycho Medisch Centrum, Leeuwarden, The Netherlands; c Department of Clinical Neurophysiology, Technical Medical Centre, University of Twente, Enschede, The Netherlands; d Department of Cognitive Neuroscience, Donders Institute for Brain, Cognition and Behaviour, Radboud University Medical Centre, Nijmegen, The Netherlands; e Department of Clinical Neurophysiology and Neurology, Medisch Spectrum Twente, Enschede, The Netherlands; f Department of Experimental Psychology, Utrecht University, Utrecht, The Netherlands

**Keywords:** Frontal alpha asymmetry, Major depressive disorder, Electroencephalogram, Trait, Personalized medicine

## Abstract

**Introduction:**

Frontal alpha asymmetry (FAA) is a proposed prognostic biomarker in major depressive disorder (MDD), conventionally acquired with electroencephalography (EEG). Although small studies attributed trait-like properties to FAA, a larger sample is needed to reliably asses this characteristic. Furthermore, to use FAA to predict treatment response, determining its stability, including the potential dependency on depressive state or medication, is essential.

**Methods:**

In the international Study to Predict Optimized Treatment in Depression (iSPOT-D), a multi-center, randomized, prospective open-label trial, 1008 MDD participants were randomized to treatment with escitalopram, sertraline or venlafaxine-extended release. Treatment response was established eight weeks after treatment initiation and resting state EEG was measured both at baseline and after eight weeks (*n* = 453).

**Results:**

FAA did not change significantly after eight weeks of treatment (*n* = 453, *p* = .234), nor did we find associations with age, sex, depression severity, or change in depression severity. After randomizing females to escitalopram or sertraline, for whom treatment response could be predicted in an earlier study, FAA after eight weeks resulted in equivalent response prediction as baseline FAA (one tailed *p* = .028).

**Conclusion:**

We demonstrate that FAA is a stable trait, robust to time, state and pharmacological status. This confirms FAA stability. Furthermore, as prediction of treatment response is irrespective of moment of measurement and use of medication, FAA can be used as a state-invariant prognostic biomarker with promise to optimize MDD treatments.

## Highlights

• Frontal alpha asymmetry (FAA) is moderately stable over time.
• Antidepressant response prediction with FAA remains consistent, even after 8 weeks of treatment.
• FAA is a differential predictor of antidepressant response robust to state and drug effects.

## 1 Introduction

Frontal alpha asymmetry (FAA) is a proposed biomarker conventionally acquired with electroencephalography (EEG). FAA has been studied for over three decades in major depressive disorder (MDD), anxiety, and other psychiatric diseases. Several studies stated, in a traditional framework of FAA, that it reflects the approach-withdrawal motivation system, i.e. the diathesis model (Davidson 1984; Harmon-Jones and Allen, 1997; Henriques and Davidson, 1991; Kelley et al., 2017). Left-sided FAA (i.e. more right-sided frontal cortical activation than left-sided) was correlated more to withdrawal behavior than to approach, which was in turn associated with a vulnerability to developing MDD. However, our meta-analysis showed that FAA cannot be used as a generic diagnostic biomarker in MDD and does not reliably differentiate MDD from non-MDD patients (Van der Vinne et al., 2017), providing evidence against the diathesis model. Only a small subgroup of severely depressed females over 53 years of age showed more right-sided alpha activity and severely depressed males over 53 years of age more left-sided alpha than control peers.

When regarding FAA as a *prognostic* rather than *diagnostic* biomarker, alpha asymmetry may be more promising. Bruder and colleagues (2008) found SSRIs (selective serotonin reuptake inhibitors) treatment responders to have more right-sided alpha asymmetry while non-responders showed opposite asymmetry, primarily over the occipital region. This was confirmed in the large international Study for Predicting Optimized Treatment – Depression sample, where specifically female SSRI responders had more right-sided FAA, and non-responders the opposite (iSPOT-D, Arns et al., 2016). To further assess properties of FAA as a prognostic biomarker, knowledge on its reliability, stability, and sensitivity to other factors, such as medication or severity of depression, needs to be established.

A predominant view in affective neuroscience is that FAA in depressed patients consists of mostly *trait*-like features, not changing over time with *state* and independent of interventions, although some studies have suggested otherwise: both longitudinal and cross-sectional designs have been used to test FAA stability (see Table 1 for a summary, and appendix Table A1 for a detailed overview of studies). With an exception of Debener et al. (2000), most studies report FAA to be stable with minor or no changes between baseline and assessment later, both in patients and healthy controls (Allen et al., 2004; Bruder et al., 2008; Davidson et al., 2003; Deldin and Chiu, 2005; Gollan et al., 2014; Keune et al., 2011; Spronk et al., 2008; Sutton and Davidson, 1997; Tomarken et al., 1992).

**Table 1:** Summary of studies on state/trait properties of frontal alpha asymmetry.

Study	Study type*	Mostly trait	Not trait - or mostly state	Subjects	EEG methods	Intervention
Allen et al., 2004	1	X		MDD, female	3 to 5 Ax., 8 or 16 weeks apart	Acupuncture
Bruder et al., 2008	1	X		MDD and HC	2 Ax., 12 weeks apart	Fluoxetine treatment
Debener et al., 2000	1		X	MDD and HC	2 Ax., 2–4 weeks apart	Several antidepressants
Deldin and Chiu, 2005	1	X		MDD and HC	4 Ax. On 1 day	Cognitive restructuring
Gollan et al., 2014	1	X		MDD and HC	2 Ax., 16 weeks apart	Behavioral activation
Keune et al., 2011	1	X		MDD	2 Ax., 8 weeks apart	Mindfulness
Spronk et al., 2008	1	X		MDD	2 Ax., pre/post-treatment	rTMS
Vuga et al., 2006	1	X		Childhood onset MDD and HC	2 Ax., 1–3.2 years apart	Some patients on ADs (13 of *n* = 49)
Davidson et al., 2003	2	X⁎⁎		HC	3 Ax., 8 weeks, 4 months	Mindfulness meditation
Hagemann et al., 2002	2	X		HC	4 Ax., all 4 weeks apart	None
Hagemann et al., 2005	2	X	X	HC	3 Ax., all 5 weeks apart	None
Sutton and Davidson, 1997	2	X		HC	2 Ax., 6 weeks apart	None
Tenke et al., 2017	2	X	X	HC	2 Ax., 5–16 days apart	None
Tomarken et al., 1992	2	X		HC	2 Ax., 3 weeks apart	None
Carvalho et al., 2011	3	X⁎⁎		MDD, remitted, and HC	1 Ax.	None
Feldmann et al., 2018	3	X⁎⁎		MDD, remitted, and HC	1 Ax.	None
Gotlib et al., 1998	3	X		MDD, remitted, and HC	1 Ax.	None
Grünewald et al., 2018	3	X⁎⁎		MDD and HC	1 Ax.	None
Nusslock et al., 2018	3	X		MDD and HC	1 Ax.	None

MDD = major depressive disorder, HC = healthy controls, Ax. = assessment(s).

^⁎^ Type 1: Multiple assessment moments with depressed patients. Type 2: Multiple assessment moments, only healthy controls. Type 3: Cross-sectional study.

⁎⁎ No explicit statements on state or trait were made by the authors (on electrode F3/F4 or F7/F8 based FAA), based on other literature we suggest our own conclusion to these results.

Cross-sectionally, several studies showed that FAA is independent of depression severity, both between patients (Allen et al., 2004; Arns et al., 2016; Feldmann et al., 2018; Gollan et al., 2014; Nusslock et al., 2018; Van der Vinne et al., 2017; Vuga et al., 2006) and within patients, including remission (Carvalho et al., 2011). This contrasts the findings by Grünewald et al. (2018) and Keune et al. (2011), where a higher level of depression complaints correlated with more left-sided FAA (albeit only in the control group of Grünewald et al.). In other cross-sectional studies on FAA stability between depressed patients and patients remitted from depression, no differences were found (Carvalho et al., 2011; Feldmann et al., 2018; Gotlib et al., 1998).

Despite some inconclusive results, the majority of findings indicate that FAA is predominantly a trait, only partially or not affected by changes in depressive state. Our meta-analysis on FAA as a diagnostic marker of depression (Van der Vinne et al., 2017) demonstrated that bias is strongly reduced from 300 cases onwards. Studies investigating FAA stability until now always studied smaller samples (*n* ≤ 85). This may explain part of the conflicting results on FAA in these studies.

This has motivated our current work that aims to replicate longitudinal results on the temporal stability of FAA by using data from the iSPOT-D dataset (baseline *n* = 1008, week-8 *n* = 453). The primary hypothesis was that FAA is reliable, and remains stable over time, with limited changes as a result of antidepressant treatment, time and state change. We therefore assessed FAA after eight weeks of antidepressant drugs and consequential state changes in mood. As age, sex, and depression severity have had a significant influence on FAA-related outcomes in iSPOT-D and other studies (e.g. Arns et al., 2016; Bruder et al., 2001; Stewart et al., 2010; Van der Vinne et al., 2017), we extended analyses by investigating possible mediation of FAA by these variables. We specifically studied MDD patients versus healthy controls differentiating subgroups identified in our previous meta-analysis, i.e. severely depressed patients over 53 years old (Van der Vinne et al., 2017). As in earlier iSPOT-D reports on FAA anxiety was not found to be of influence, we did not add this variable to our analyses.

For clinical use of FAA as a biomarker for treatment response, it is relevant to assess stability and robustness to medication. Stability is particularly an advantage when patients are already on an AD preceding baseline (that often have long half-life times requiring wash-out periods of weeks) and FAA remains unaffected. We therefore also assess outcome prediction with FAA recorded after eight weeks treatment. In our previous report (Arns et al., 2016), at baseline, right-sided FAA in females was associated with favorable outcome to the SSRIs escitalopram and sertraline, whereas left-sided FAA was not. If FAA *is* prognostic for AD treatment outcome in specific subsamples, and FAA is indeed a stable *trait*, FAA after eight weeks on an AD should still be able to predict treatment outcome for females in agreement with our previous study (Arns et al., 2016). We hypothesized that analysis of week-8 medicated EEG data would result in the same treatment prediction results as baseline unmedicated data did.

## 2 Materials and methods

### 2.1 Design

This is an international multi-center, randomized, prospective open-label trial (Phase-IV clinical trial), in which MDD patients were randomized to escitalopram, sertraline, or venlafaxine-XR treatment in a 1:1:1 ratio. The study protocol details, including a power calculation, have been published by Williams et al. (2011). This design was deliberately chosen to mimic real-world practice with the aim of optimizing the translatability to real world settings.

### 2.2 MDD patients and treatment

**Fig 1:**
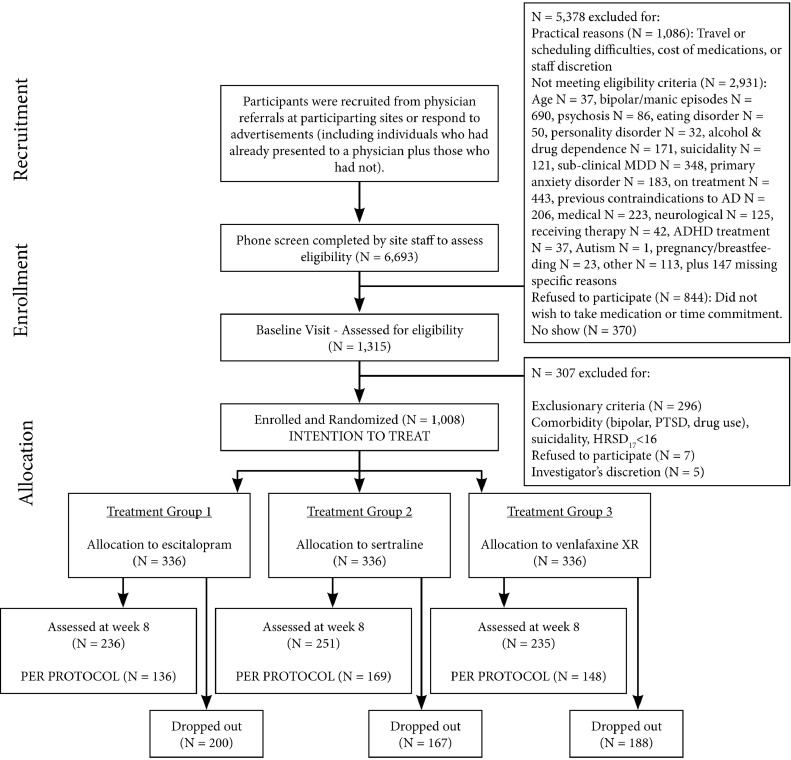
Consort diagram of the iSPOT-D study. *Abbreviations*: ADHD, attention deficit hyperactivity disorder; AD, antidepressant treatment; HRSD_17_, 17-item Hamilton rating scale for depression; MDD, major depressive disorder; PTSD, post-traumatic stress disorder; XR, extended release.

We included 1008 MDD patients, recruited between October 2008 and January 2011. A detailed description of the study assessments, inclusion/exclusion criteria, diagnostic procedures and treatment is available in Williams et al. (2011). In summary, the primary diagnosis of nonpsychotic MDD was confirmed before randomization using the Mini-International Neuropsychiatric Interview (MINI-Plus, Sheehan et al., 1998), according to DSM-IV criteria, and a score ≥16 on the 17-item Hamilton Rating Scale for Depression (HRSD_17_). Additional measuring of depression complaints was done with the Very Quick Inventory of Depressive Symptomatology – Self Report (VQIDS-SR_5_, De La Garza, John Rush, Grannemann, and Trivedi, 2017). Comorbid anxiety disorders were allowed (present in 6.2% [specific phobia] to 10.5% [social phobia] of patients). All patients were either medication-naive or, if previously prescribed an antidepressant medication, had undergone a washout period of at least five half-lives before the baseline visit clinical and EEG assessments. After the baseline visit, patients were randomized to one of three antidepressant medication treatments. After eight weeks of treatment, patients were tested again using the HRSD_17_, the VQIDS-SR_5_ and an EEG assessment (Fig. 1). This study was approved by the institutional review boards at all of the participating sites and this trial was registered with ClinicalTrials.gov. Registration number: NCT00693849; URL: http://clinicaltrials.gov/ct2/show/NCT00693849.

### 2.3 Pre-treatment assessments

EEG recordings were performed using a standardized methodology and platform (Brain Resource Ltd., Australia). Details of this procedure (Arns et al., 2008; Williams et al., 2011) and of its reliability and across-site consistency have been published elsewhere (Paul et al., 2007; Williams et al., 2005). In summary, subjects were seated in a sound and light attenuated room that was controlled at an ambient temperature of 22 °C. EEG data were acquired from 26 channels: Fp1, Fp2, F7, F3, Fz, F4, F8, FC3, FCz, FC4, T3, C3, Cz, C4, T4, CP3, CPz, CP4, T5, P3, Pz, P4, T6, O1, Oz and O2 (Quik-cap; NuAmps; 10–20 electrode international system). EEG was assessed for two minutes with eyes open (EO) (with the subject asked to fixate on a red dot on the screen) and two minutes with eyes closed (EC). The subject was instructed to remain relaxed for the duration of the recording. The operator did not intervene when drowsiness patterns were observed in the EEG. Data were referenced to averaged mastoids with a ground at AFz. Horizontal eye movements were recorded with electrodes placed 1.5 cm lateral to the outer canthus of each eye. Vertical eye movements were recorded with electrodes placed 3 mm above the middle of the left eyebrow and 1.5 cm below the middle of the left bottom eyelid. Skin resistance was <5 K Ohms for all electrodes. The sampling rate of all channels was 500 Hz. A low pass filter with an attenuation of 40 dB per decade above 100 Hz was employed prior to digitization.

### 2.4 EEG analysis

A detailed overview of the data-analysis can be found in Arns et al. (2016). In summary, data were (1) filtered (0.3–100 Hz and notch); (2) EOG-corrected using a regression-based technique similar to that used by Gratton et al. (1983), segmented in 4-second epochs (50% overlapping), and an automatic de-artifacting method was applied. This EEG processing pipeline was also validated against an independent manual-processing pipeline (Arns et al., 2016). For further analysis, an average reference was applied, data were filtered (alpha power (µV^2^): 8–13 Hz) and FAA was calculated between F3 and F4 as (F4 – F3)/(F4 + F3).

### 2.5 Statistics

Normal distribution was inspected, and appropriate transformations performed in case of non-normality. Non-log transformed alpha power was used to calculate FAA. Remission was defined as a score ≤7 on the HRSD_17_ eight weeks after starting treatment (current endpoint), and response was defined as *a* ≥ 50% decrease in HRSD_17_ score from baseline to eight weeks. To control for antidepressant side-effects, we employed the VQIDS-SR_5_, developed specifically to focus on the core symptoms of depression. This enabled us to measure true depression severity, ruling out antidepressant side-effects such as physical complaints. We repeated ANOVAs from paragraph 3.2 and 3.3 and replaced all HRSD_17_ variables with VQIDS-SR_5_ equivalents. Results are reported in Appendix D.

Differences in age, sex, education, and depression severity at baseline were tested using one-way ANOVA or non-parametric tests, depending on its distribution. We only included patients who returned for their week-8 visit while on their assigned medication, having followed this treatment for a minimum of 6 weeks (‘per-protocol’ grouping, also see the Consort diagram in Fig. 1).

FAA reliability analysis was performed by calculating Intraclass Correlations (ICCs) across baseline and week-8 measurements. A full-factorial Repeated Measures ANOVA was conducted with the within–subject factor FAA Change Eyes Closed (FAA at baseline and after eight weeks) and between-subject factor Treatment arm (comparing drug effects of respectively escitalopram, sertraline, and venlafaxine). Given the large sample size we set the significance level for main effects found for FAA Change in the main analyses at *p* ≤ .01, for interaction effects this remained at a conventional level of *p* ≤ .05. When significant interactions were found prompting subgroup analyses, again a level of *p* ≤ .05 was used. Effect sizes (ES) of main effects are reported in Cohen's *d*. FAA stability was also tested through Pearson correlations between FAA Change and HRSD_17_ Change.

Post hoc, we repeated the Repeated Measures and Pearson correlations analyses in the subgroups of moderately and severely depressed (HRSD_17_ score of ≥24) over the age of 53, separately for males and females (conform our meta-analysis, Van der Vinne et al., 2017). However, as these groups might lead to underpowered tests, we also performed a custom Repeated Measures ANCOVA on the whole dataset, now also including covariates Age and Depression severity, separately for males and females.

When a null hypothesis was not rejected by any of the ANOVAs or correlational analyses, we utilized Bayesian alternatives. This was done for testing evidence of *absence* of a change in FAA, using the Bayesian Repeated Measures ANOVA framework (based on work by Jeffreys (1961) and Rouder et al. (2009)). We analyzed the data with JASP (JASP Team, 2017). The first null hypotheses states that there is no difference in FAA between baseline and after 8 weeks. The second that FAA Change is not correlated to HRSD_17_ Change. The two-sided alternative hypotheses state that FAA changed after eight weeks, or that FAA is correlated to HRSD_17_ Change.

Through a Repeated Measures model (Arns et al., 2016), we again predicted treatment outcome in females taking an SSRI (escitalopram or sertraline), while this time replacing baseline FAA with week-8 FAA (within subjects variable FAA Condition (EC and EO), and between subjects variable Response, and covariate Age). We tested effects one-tailed (halved *p*-values were reported) because we specifically expected more right-sided FAA in SSRI responders than in non-responders, implying that a result in the unexpected direction would lead to the same conclusion as finding no differences at all (Ruxton and Neuhäuser, 2010). In Appendix B, we explain why we compare the smaller sample containing only patients who were present for the assessment after 8 weeks, to the larger sample with *all* baseline patients from the previous study.

## 3 Results

Of the 1008 MDD patients enrolled, the final MDD sample for the FAA Change analyses consisted of 453 MDD patients. The remaining 555 patients were left out of the study: they either never started treatment, had less than 6 weeks of medication, or had no week-8 assessment (or it was of insufficient quality) (see Fig. 1). Table 2 shows demographic information and response and remission rates for included patients. There were no differences between the three treatment groups regarding age, sex, baseline MDD, anxiety severity, remission and response rates, or number of rejected EEG epochs. Approximately 5.3% of EEG epochs were rejected due to artifacts for the MDD group during EC.

**Table 2:** Demographic features and treatment outcomes for patients who completed treatment.

	Escitalopram	Sertraline	Venlafaxine-XR	Total
N	136	169	148	453
Females	71	96	80	247
% Female	52.5	56.8	54.1	54.5
Average age (years)	38.27	38.72	37.98	38.34
HRSD_17_ baseline	21.45	21.74	21.45	21.56
HRSD_17_ week-8	8.62	9.25	9.01	8.98
VQIDS-SR_5_ baseline	8.01	8.34	7.99	8.13
VQIDS-SR_5_ week-8	3.26	3.35	3.21	3.28
% Remission (HRSD_17_)	51.5	46.7	44.6	47.5
% Response (HRSD_17_)	66.2	66.9	66.2	66.4

### 3.2 FAA change over time

ICCs for FAA with both continuous and dichotomous (leftward or rightward FAA) variables were 0.276 and 0.256, respectively. The Repeated Measures ANOVA revealed no evidence for change in FAA after AD treatment (*F*(1,450) = 1.421, *p* = .234), nor an interaction with Treatment Arm (*F*(2,450) = 0.690, *p* = .502). FAA Change was neither significantly correlated to the change score in HRSD_17_ (*r* = 0.039, *p* = .410), nor to the percentage change in HRSD_17_ (*r* = 0.047, *p* = .323).

Results of Bayesian Repeated Measures testing of invariant (constant) FAA revealed a Bayes factor indicating evidence for the null hypothesis. The models with the factors FAA Change and Treatment Arm showed that the data occur >7.4 times more likely under the null hypothesis, than under any alternative model with (a combination of) the factors. Bayesian Pearson correlations between FAA Change and the difference score HRSD_17_/the percentage difference of HRSD_17_ reveal moderate to strong results. The data are respectively 12.1 and 9.3 times more likely to occur under the null hypothesis than under the model assuming a correlation between the variables. See Appendix F for an elaboration on results and JASP tables.

### 3.3 Extended repeated measures model and correlations

Focusing on variables known to have an influence on FAA, specifically in the subgroup we thought to be prone to changes in FAA (severely depressed females and males over 53 years old), we did not find significant changes, although subsample sizes were small. Furthermore, in these subgroups the FAA Change score was not significantly correlated to the change score in HRSD_17_ (see appendix Table C1 for all statistics). Bayesian Repeated Measures ANOVAs for the two sex groups of severely depressed over the age of 53 reveal anecdotal (i.e. worth no more than a bare mention, a customary description for BFs ranging 1–3) to moderate results. Most models therefore provided no conclusive evidence for either the null or the alternative hypotheses, although some models indicated moderate evidence of the data being more likely to occur under the null hypothesis. See Appendix F for an elaboration on results and JASP tables.

Extending the Repeated Measures model from paragraph 3.2 showed that - irrespective of sex - baseline severity and age are not significantly contributing to FAA Change. Bayesian Repeated Measures alternatives for the extended ANOVAs showed similar results to paragraph 3.2. For females, the data are ≥6.6 times more likely to occur under the null hypothesis, than under any alternative model with (a combination of) the factors, and ≥4.7 times more likely in case of males. See Appendix F for an elaboration on results and JASP tables.

### 3.4 Treatment prediction using medicated week-8 data in females

Treatment outcome prediction with week-8 data, revealed a similar prediction pattern as baseline data reported in Arns et al. (2016): one-tailed testing of the prediction of response in females taking an SSRI for depression (escitalopram or sertraline), treatment response effects remained significant with week-8 FAA on group level (*F*(1,150) = 3.725, *p* = .028). Furthermore, the response effect of FAA was again lacking after eight weeks in the venlafaxine group.

**Fig 2:**
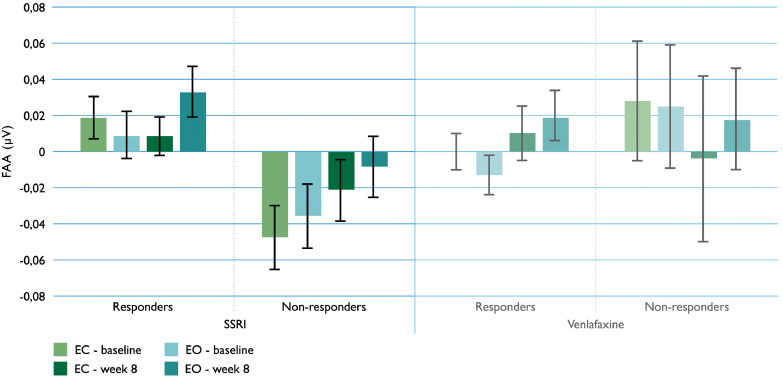
Mean values of female frontal alpha asymmetry (FAA, eyes open and eyes closed [EO and EC]), for the SSRI and venlafaxine groups, split up for responders and non-responders. Error bars represent standard error of the mean. The means and error bars indicate that baseline and week-8 FAA were not significantly different in predicting treatment outcome in females; SSRI responders showed right-sided, non-responders left-sided FAA. No differences were, yet again, observed for the venlafaxine group. The equivalent of this data for males is available in Appendix G.

The week-8 SSRI data in Fig. 2 visualize how responders were significantly more right-sided than non-responders (based on female FAA means reported in appendix Table E1). Fig. 2 also shows how the response effect was similar to the baseline assessment. This was despite the confidence interval (CI) of FAA in Fig. 2 (SSRI non-responders) showing no significant difference from 0 when measured with EO after eight weeks. No interactions with age were observed. The equivalent of Fig. 2 data for males is available in Appendix G.

Cohen's *d* comparing FAA change scores of female SSRI responders and non-responders was 0.304. When using the direction of week-8 FAA alone to prescribe an SSRI or SNRI would have improved the overall remission rate from 47% to 56–58% for an SSRI.

## 4 Discussion

We investigated the stability of FAA in MDD patients during antidepressant treatment. We hypothesized that FAA is a robust metric, insensitive to time, antidepressant drug treatment and state changes. FAA did not change significantly after eight weeks of escitalopram, sertraline, or venlafaxine treatment, despite a relatively low reliability of the FAA measurements. Additional Bayesian testing revealed that a stable FAA is more likely than a change in FAA over time after antidepressant treatment. Furthermore, post-hoc tests with variables known to have influence on FAA (in earlier iSPOT-D studies), revealed no differential temporal changes in FAA in depressed patients differing on age, sex, depression severity, or change in depression severity. Focusing on core depression symptoms only (as measured by the VQIDS-SR_5_, see appendix D), we found similar results.

To further confirm FAA temporal stability, we hypothesized that predicting treatment outcome in females taking SSRIs would lead to similar outcome when using *week-8* FAA instead of the previously studied *baseline* FAA (Arns et al., 2016). This re-analysis indeed confirmed an overall response in the SSRI group with right-sided FAA, and a non-response with left-sided FAA. Although the effect size was less pronounced with week-8 data, week-8 FAA yielded the same conclusions as the baseline measurements, with a Cohen's *d* of 0.547 in the previous analyses vs. our current 0.304. Furthermore, we yielded the same improvement in remission rates when week-8 FAA had been used for ‘prescribing’ medication: previous SSRI remission rates improved from 46% to 53–60% using baseline FAA, the current from 47% to 56–58% using week-8 FAA. This extends the use of FAA as a prognostic biomarker, as response prediction was neither modified by moment of assessment, nor by AD treatment.

The low reliability was unexpected, and implies that FAA following treatment was not as stable as in previous studies. In several studies, FAA was found to be relatively reliable and consistent, based on ICCs and Cronbach's alpha (Allen et al., 2004; Debener et al., 2000; Keune et al., 2011; Sutton and Davidson, 1997; Towers and Allen, 2009). Especially Towers and Allen (2009) demonstrated FAA consistency, through several methods. An important difference is the use of a single FAA statistic per assessment time (two in total) in our study vs. several other studies using (fictive) multiple time points. This could account for our lower reliability. Despite the low ICC, we did replicate no evidence for a significant change in FAA over time, in a large sample (*N* = 453).

To our knowledge, this is the first study to assess the temporal stability of FAA in a large sample. This supports previous studies showing that FAA mainly depends on a considerable number of trait-like features, insensitive to antidepressant treatment, age, sex or depression severity (Allen et al., 2004; Arns et al., 2016; Bruder et al., 2008; Carvalho et al., 2011; Deldin and Chiu, 2005; Feldmann et al., 2018; Gollan et al., 2014; Keune et al., 2011; Nusslock et al., 2018; Spronk et al., 2008; Sutton and Davidson, 1997; Tomarken et al., 1992; Van der Vinne et al., 2017; Vuga et al., 2006). Similarly, Segrave et al. (2011) showed no evidence for antidepressant elicited changes in FAA when comparing a small group of depressed patients on ADs with unmedicated patients. In other small cohorts, FAA was not modified by the use of antidepressive medication either (Bruder et al., 2008; Vuga et al., 2006), in agreement with our observations.

In the prevailing approach-withdrawal motivation system hypothesis, it is assumed that FAA is associated with lifetime MDD (having had at least one depressive episode in one's life), and not specifically current MDD. This is an important distinction, and our results initially support this theory. The motivation system hypothesis states that FAA is not expected to change as a result of changes in MDD status, and ultimately not with MDD remission. However, with establishing FAA (in)stability, our study would neither provide evidence for, nor against the theory. That is, if we would have found the opposite result (a change in FAA), this could have been explained as well, by the related capability model (Coan et al., 2006). This model states that resting state FAA is more prone to fluctuations than FAA measured after inducing positive or negative mood. Because we measured resting state FAA, either outcome could be explained within the approach-withdrawal motivation system, given the capability model. Therefore, it is difficult to unambiguously place our results in the existing theories. Note that our earlier findings were less compatible with the motivation system: Firstly, in the approach-withdrawal motivation system, left-sided FAA is theorized to be more associated with withdrawal behavior and depression. But brain asymmetry was found not to be different in these groups as measured both through EEG FAA (Van der Vinne et al., 2017), and through fMRI in a recent large ENIGMA consortium study (de Kovel et al., 2019). Secondly, prognostic results for females in the FAA iSPOT-D study (Arns et al., 2016) revealed heterogeneity in MDD patients, not consistent with assuming a homogenic FAA related vulnerability for MDD. In sum, the current study was not designed to directly investigate the approach-withdrawal motivation theory, and cannot provide support in favor of or against the theory.

We show that FAA is a robust metric, suitable for sex specific treatment prediction under challenging circumstances, such as state, time, the use of common antidepressive agents and drug changes. This suggests reliable implementation in clinical practice as a prognostic biomarker in both medicated and unmedicated patients.

## 5 Conclusions

In an adequately powered sample, we demonstrate that (1) neither antidepressant medication, (2) nor MDD state and severity, have systematic effects on FAA. This confirms FAA stability. Furthermore, as prognosis of treatment response is irrespective of the moment of measurement, FAA may serve as a robust biomarker to optimize MDD treatments.

## Figures and Tables

**Table A1:** Overview of FAA stability related studies.

Study	Study type*	*N*=	Subjects	EEG Methods	Intervention	Relevant factors
Allen et al., 2004	1	30	MDD (females)	3 to 5 Ax., 8 or 16 weeks apart	Acupuncture (specific and non-specific)	HRSD change score
Bruder et al., 2008	1	18	MDD and HC	2 Ax., 12 weeks apart	Fluoxetine treatment	Response (“CGI-I rating much or very much improved”)
Debener et al., 2000	1	15 and 22	MDD and HC	2 Ax., 2–4 weeks apart	Several antidepressants	BDI-score
Deldin and Chiu, 2005	1	15 and 18	MDD and HC	4 Ax. On 1 day	Cognitive restructuring	Happiness change score
Gollan et al., 2014	1	37 and 35	MDD and HC	2 Ax., 16 weeks apart	Behavioral activation	IDS-SR
Keune et al., 2011	1	78	MDD	2 Ax., 8 weeks apart (neutral vs. sad state)	Mindfulness	BDI en BDI-Change, gender
Spronk et al., 2008	1	8	MDD	2 Ax., pre/post-treatment	rTMS	None that was associated with frontal asymmetry
Vuga et al., 2006	1	49 and 50	Childhood onset depression and HC	2 Ax., 1–3.2 years apart	Some cases on ADs (13 of *n* = 49)	Age, sex, BDI
Davidson et al., 2003	2	41	HC	2 Ax., 8 weeks, 4 months	Mindfulness meditation	
Hagemann et al., 2002	2	59	HC	4 Ax., all 4 weeks apart	None	
Hagemann et al., 2005	2	59	HC	3 Ax., all 5 weeks apart	None	
Sutton and Davidson, 1997	2	46	HC	2 Ax., 6 weeks apart	None	No depression scores (only BIS/BAS and PANAS)
Tenke et al., 2017	2	39	HC	2 Ax., 5–16 days apart	None	
Tomarken et al., 1992	2	85	HC	2 Ax., 3 weeks apart	None	
Carvalho et al., 2011	3	12, 8 and 7	MDD, remitted and HC	1 Ax.	None	BDI
Feldmann et al., 2018	3	22, 16 and 34	MDD, remitted and HC (also other groups, with comorbid anxiety)	1 Ax.	None	BDI
Gotlib et al., 1998	3	16, 31 and 30	MDD, remitted and HC	1 Ax.	None	
Grünewald et al., 2018	3	28 and 31	MDD and HC	1 Ax.	None	BDI
Nusslock et al., 2018	3	37 and 69	MDD and HC	1 Ax.	None	BDI

Study	Relevant analyses	Calculation FAA	Conclusion
Allen et al., 2004	FAA solely: ICCs. FAA in relation to symptoms: Correlations & multivariate Repeated Measures analysis of variance (HRSD-score) with changing covariates (3 asymmetry measures)	ln[Right] - ln[Left]	Stable across time and independent of depression severity.
Bruder et al., 2008	1. Interaction Response-Nonresponse-HC * Hemisphere * Site. 2. Interaction Response * Site * Hemisphere * Session. 3. Test-retest correlations.	Interaction of site and hemisphere	Overall stable. 1. No FAA differences between groups. 2. No change of FAA over time. 3. Moderate test-rest correlations of FAA after treatment.
Debener et al., 2000	1. Cronbachs alpha FAA for internal consistency. 2. Correlation FAA-BDI. 3. Temporal stability through Pearson correlations.	1. Interaction of session * site * region (posterior-anterior) * Hemisphere 2. ln(right) - ln(left)	Overall: Not a stable measure. 1. Good internal consistency. 2. No correlation with BDI, so state-independent. 3. Unstable temporal stability of frontal regions (not posterior).
Deldin and Chiu, 2005	1. Interaction Diagnosis * Block * Region * Laterality 2. Correlation FAA-Happiness score	1. Interaction with region and laterality 2. ln F4 - ln F3	Overall: no changes between assessments on the same day. 2. No correlation.
Gollan et al., 2014	1. Rep Measures ANOVA FAA over time. 2. Correlation pre FAA-pre IDS/pre FAA-post IDS/post FAA-pre IDS/post FAA-post IDS	log F4 - log F3	Overall: FAA = stable, trait-like. 1.No changes in FAA. 2. No significant correlations.
Keune et al., 2011	1. Cronbachs alpha. 2. ANOVA interaction FAA time 1 * time 2. 3. Correlation FAA-affect scores (to test state). 4. Test-retest reliability with Pearson product moment correlations. 5. Correlation FAA-BDI time 1. 6. Correlation FAA Change-BDI Change. 7. Interactions with gender	subtracting power values in the left hemisphere from the values in the right hemisphere	1. Stable. 2. Change (in sad condition, not in neutral). 3. No correlation with affect, so stable. 4. Correlates, so reliable. 5. Significant correlation FAA-BDI in sad condition (not in neutral, this was only for other sites). 6. No correlation. 7. No gender interaction effects.
Spronk et al., 2008	Interaction of Time * Hemisphere (left: F3, FC3, F7. Right: F4, FC4, F8)	Interaction of time and hemisphere	No interaction for alpha1, alpha2 and alpha. Alpha seems a trait.
Vuga et al., 2006	1. Cronbach's alpha. 2. ANOVA with Age, apart for the sexes. 3. ICC 4. ANOVA with group, sex, and FAA at Time 2 as dependent variable, on Time 1 as dependent variable. 5. Same analyses, apart for with and without medication. 6. Regression on FAA Time 2, with FAA Time 1, BDI and BDI-change.	ln(F4) - ln(F3)	Overall: Moderate to high long term stability. 1. Consistent. 2. No influence of Age or Sex. 3. Moderately stable. 4. Stable after p-value correction, without correction there would be differences. 5. Same results with and without medication, so stable. 6. Depressive symptom severity and change in symptoms did not affect EEG asymmetry stability.
Davidson et al., 2003	Group (mindfulness/waitlist) * Time.	ln(F4) – ln(F3), ln(F8) – ln(F7)	Changes in asymmetry were only observed in other electrode pairs, not in F3/4 or F7/8.
Hagemann et al., 2002	Model of LST theory	ln power density F4 − ln power density F3	60% trait - 40% state
Hagemann et al., 2005	Model of LST theory	ln F4 − ln F3	40–50% state
Sutton and Davidson, 1997	Averaged over 13 asymmetry measures (from 13 homologous electrode pairs): Cronbach's alpha (0.87) and ICC (0.57)	log F4 − log F3	High to modest test-retest reliability
Tenke et al., 2017	Test-retest correlations	F4 − F3	Variation in outcome: low in general, but it is correlating. Test-retest correlations differ per research site, but is in general significant for F3-F4: 0.371 (see supplement).
Tomarken et al., 1992	1. t-tests of asymmetry measures. 2. ICCs and Pearson correlations. 3. Cronbach's alphas	log R minus log L power density	1. Stable: "…asymmetry measures tended to be associated with nonsignificant shifts in mean values over time". 2. Not very high, buy significant ICCs (0.66 for Avg Ref baseline only, 0.79 for Avg Ref across time). 3. Acceptable-to-excellent Cronbach's alphas.
Carvalho et al., 2011	1. ANOVA on FAA, with hemisphere and group. 2. Correlation FAA-BDI.	ln[right] − ln[left]	Overall: doubtful, whether cross-sectional data is sufficient to establish trait properties. But there are no indications against FAA having trait properties. 1. No interaction especially between MDD and remitted, but also controls. 2. No correlation.
Feldmann et al., 2018	1. One-way ANOVAs on FAA between the HC, Mda- and rMDa- . 2. Correlation FAA-BDI	ln[right ROI] − ln[left ROI]	The most relevant results: 1. No differences between MDD and remitted. 2. No correlations.
Gotlib et al., 1998	Regression with 1st predictor “Never vs. Ever depressed” and 2nd predictor “Currently depressed vs. remitted”	log R − log L	Most relevant results: No difference between currently depressed and remitted. FAA seems to be a state independent marker.
Grünewald et al., 2018	Correlation FAA-BDI	ln[right] − ln[left]	Overall no specific conclusion on state or trait, but no correlations were found in the MDD group.
Nusslock et al., 2018	Correlation FAA-BDI	right − left	Overall: "[our results] highlight the trait like quality of reduced relative left frontal EEG activity." 1. No correlation.

*Type 1: Multiple assessment moments with depressed patients. Type 2: Multiple assessment moments, only healthy controls. Type 3: Cross-sectional study. MDD = major depressive disorder, HC = healthy controls, Ax. = assessment(s), HRSD = Hamilton Rating Scale for Depression, CGI = Clinical Global Impression, BDI = Beck Depression Inventory, IDS-SR = Inventory of Depressive Symptomatology-Self Report, BIS/BAS = Behavioral Avoidance/Inhibition Scales, PANAS = Positive and Negative Affect Scale, ICC = Intraclass Correlation Coefficient, HRSD = Hamilton Rating Scale for Depression, HC = healthy controls, BDI = Beck Depression Inventory, LST theory = latent state-trait theory, Avg Ref = average reference.

**Table B1:** P-values of mentioned interaction effects in the re-analysis of Arns et al. (2016) with data only of MDD patients who had measurements after 8 weeks (thus excluding FAA baseline measurements of patients who did not return for follow-up).

	Original analysis	Original analysis without patients with no follow-up	*Re*-analysis with week-8 FAA*
Females SSRI: Response	*P* = .001	*P* = .001	*P* = .028
Females venlafaxine: Response	*P* = .070	*P* = .011	*P* = .821

**Table C1:** Statistics paragraph 3.3. A: Severely depressed ≥53 years old only. B: Whole dataset.

	Sex	(Interaction) Effect	F (df)	p (F)	r	p (r)
A	Females	FAA Change	2.080 (1,14)	.171		
		FAA Change * Treatment arm	2.425 (2,14)	.125		
	Males	FAA Change	0.092 (1,7)	.771		
		FAA Change * Treatment arm	0.061 (2,7)	.941		
	Females	FAA Change * HRSD_17_ Change			0.259	.316
	Males	FAA Change * HRSD_17_ Change			−0.070	.849
B	Females	FAA Change	0.355 (1,235)	.552		
		FAA Change * Treatment arm	0.714 (2,235)	.491		
		FAA Change * Age	0.889 (1,235)	.344		
		FAA Change * Depression severity	0.645 (1,235)	.423		
		FAA Change * Treatment arm * Age	0.849 (2,235)	.429		
		FAA Change * Treatment arm * Depression severity	0.846 (2,235)	.430		
		FAA Change * Age * Depression severity	1.254 (1,235)	.264		
		FAA Change * Treatment arm * Age * Depression severity	1.148 (2,235)	.319		
	Males	FAA Change	0.029 (1,194)	.864		
		FAA Change * Treatment arm	0.282 (2,194)	.755		
		FAA Change * Age	0.024 (1,194)	.878		
		FAA Change * Depression severity	0.022 (1,194)	.881		
		FAA Change * Treatment arm * Age	0.292 (2,194)	.747		
		FAA Change * Treatment arm * Depression severity	0.471 (2,194)	.625		
		FAA Change * Age * Depression severity	0.052 (1,194)	.820		
		FAA Change * Treatment arm * Age * Depression severity	0.352 (2,194)	.704		

**Table D1:** VQIDS-SR_5_ Statistics paragraph 3.3. A: Severely depressed ≥53 years old only. B: Whole dataset.

	Sex	(Interaction) Effect	F (df)	p (F)	r	p (r)
A	Females	FAA Change * VQIDS Change			−0.121	.644
	Males	FAA Change * VQIDS Change			0.127	.381
B	Females	FAA Change	0.530 (1,225)	.467		
		FAA Change * Treatment arm	0.002 (2,225)	.998		
		FAA Change * Age	0.930 (1,225)	.336		
		FAA Change * VQIDS Depression severity	0.125 (1,225)	.724		
		FAA Change * Treatment arm * Age	0.066 (2,225)	.936		
		FAA Change * Treatment arm * VQIDS Depression severity	0.145 (2,225)	.865		
		FAA Change * Age * VQIDS Depression severity	0.384 (1,225)	.536		
		FAA Change * Treatment arm * Age * VQIDS Depression severity	0.351 (2,225)	.705		
	Males	FAA Change	0.991 (1,225)	.321		
		FAA Change * Treatment arm	1.491 (2,225)	.228		
		FAA Change * Age	0.407 (1,225)	.524		
		FAA Change * VQIDS Depression severity	1.214 (1,225)	.272		
		FAA Change * Treatment arm * Age	0.773 (2,225)	.463		
		FAA Change * Treatment arm * VQIDS Depression severity	1.739 (2,225)	.179		
		FAA Change * Age * VQIDS Depression severity	0.654 (1,225)	.420		
		FAA Change * Treatment arm * Age * VQIDS Depression severity	1.158 (2,225)	.316		

**Table E1:** FAA means of the different subgroups reported in paragraph 3.5. Split on sex, medication type, EEG condition, response group, and time of assessment.

			Baseline		Week 8	
Sex	Medication type	EEG condition*	Response	Non-response	Response	Non-response
Female	SSRI	EC	0.019	−0.048	0.009	−0.022
		EO	0.009	−0.036	0.033	−0.008
	SNRI	EC	0.000	0.028	0.010	−0.004
		EO	−0.013	0.025	0.020	0.018
Male	SSRI	EC	0.003	0.017	0.013	0.030
		EO	0.015	0.036	0.044	0.036
	SNRI	EC	−0.015	−0.028	−0.031	−0.023
		EO	−0.010	−0.045	−0.036	0.002

*EC = eyes closed, EO = eyes open.

**Table F1:** Bayesian Repeated Measures ANOVA main analysis.

Model comparison					
Models	P(M)	P(M|data)	BF_M_	BF_01_	error%
Null model (incl. subject)	.200	.856	23.749	1.000	
FAA Change	.200	.114	0.517	7.483	1.276
Treatment	.200	.026	0.107	32.853	0.604
FAA Change + Treatment	.200	.004	0.014	240.356	2.282
FAA Change + Treatment + FAA Change *Treatment	.200	1.675e-4	6.702e-4	5109.119	2.471

Note: All models include subject.

**Table F1:** Continued. Bayesian Repeated Measures ANOVA main analysis.

Analyses of effects			
Effects	P(incl)	P(incl|data)	BF_Inclusion_
FAA Change	.400	.118	0.134
Treatment	.400	.030	0.031
FAA Change *Treatment	.200	1.675e-4	0.047

Note: Compares models that contain the effect to equivalent models stripped of the effect. Higher-order interactions are excluded.

**Table F2:** Bayesian Pearson correlations FAA Change vs. HRSD_17_ Change/HRSD_17_% Change.

			r	BF_01_
FAA Change	–	HRSD_17_ Change	0.039	12.111
FAA Change	–	HRSD_17_% Change	0.052	9.275

**Table F3:** Bayesian Repeated Measures ANOVA for severely depressed males ≥53 years old.

Model comparison					
Models	P(M)	P(M|data)	BF_M_	BF_01_	error%
Null model (incl. subject)	.200	.363	2.282	1.000	
FAA Change	.200	.175	0.851	2.070	0.701
Treatment	.200	.269	1.472	1.351	0.687
FAA Change + Treatment	.200	.134	0.618	2.715	1.744
FAA Change + Treatment + Time*Treatment	.200	.059	0.249	6.195	2.327

Note: All models include subject.

**Table F3:** Continued. Bayesian Repeated Measures ANOVA for severely depressed males ≥53 years old.

Analyses of effects			
Effects	P(incl)	P(incl|data)	BF_Inclusion_
FAA Change	.400	.309	0.489
Treatment	.400	.403	0.748
FAA Change *Treatment	.200	.059	0.438

Note: Compares models that contain the effect to equivalent models stripped of the effect. Higher-order interactions are excluded.

**Table F4:** Bayesian Repeated Measures ANOVA for severely depressed females ≥53 years old.

Model comparison					
Models	P(M)	P(M|data)	BF_M_	BF_01_	error%
Null model (incl. subject)	.200	.393	2.592	1.000	
FAA Change	.200	.211	1.069	1.864	1.400
Treatment	.200	.171	0.825	2.299	0.528
FAA Change + Treatment	.200	.091	0.402	4.304	0.922
FAA Change + Treatment + FAA Change *Treatment	.200	.134	0.617	2.944	1.372

Note: All models include subject.

**Table F4:** Continued. Bayesian Repeated Measures ANOVA for severely depressed females ≥53 years old.

Analyses of effects			
Effects	P(incl)	P(incl|data)	BF_Inclusion_
FAA Change	.400	.302	0.536
Treatment	.400	.262	0.434
FAA Change *Treatment	.200	.134	1.462

Note: Compares models that contain the effect to equivalent models stripped of the effect. Higher-order interactions are excluded.

**Table F5:** Bayesian Repeated Measures ANOVA for females, with factors and covariates Treatment Arm, Age and Baseline HRSD17.

Model comparison					
Models	P(M)	P(M|data)	BF_M_	BF_01_	error%
Null model (incl. subject)	.050	.547	22.983	1.000	
FAA Change	.050	.083	1.720	6.596	1.069
Age	.050	.092	1.935	5.922	1.199
FAA Change + Age	.050	.014	0.268	39.377	1.598
Baseline HRSD_17_	.050	.097	2.036	5.657	1.928
FAA Change + Baseline HRSD_17_	.050	.015	0.286	36.858	1.939
Age + Baseline HRSD_17_	.050	.027	0.534	20.007	1.962
FAA Change + Age + Baseline HRSD_17_	.050	.004	0.077	134.758	2.073
Treatment	.050	.073	1.490	7.526	0.651
FAA Change + Treatment	.050	.011	0.216	48.653	1.854
Age + Treatment	.050	.013	0.243	43.438	1.488
FAA Change + Age + Treatment	.050	.002	0.039	268.859	4.110
Baseline HRSD_17_ + Treatment	.050	.013	0.255	41.259	1.331
FAA Change + Baseline HRSD_17_ + Treatment	.050	.002	0.040	263.804	1.689
Age + Baseline HRSD_17_ + Treatment	.050	.004	0.076	137.616	3.325
FAA Change + Age + Baseline HRSD_17_ + Treatment	.050	5.979e-4	0.011	915.659	1.734
FAA Change + Treatment + FAA Change*Treatment	.050	.001	0.022	472.071	5.124
FAA Change + Age + Treatment + FAA Change*Treatment	.050	1.915e-4	0.004	2858.225	2.712
FAA Change + Baseline HRSD_17_ + Treatment + FAA Change*Treatment	.050	2.204e-4	0.004	2483.772	8.576
FAA Change + Age + Baseline HRSD_17_ + Treatment + FAA Change*Treatment	.050	5.817e-5	0.001	9410.129	2.373

Note: All models include subject.

**Table F5:** Continued. Bayesian Repeated Measures ANOVA for females, with factors and covariates Treatment Arm, Age and Baseline HRSD17.

Analyses of effects			
Effects	P(incl)	P(incl|data)	BF_Inclusion_
FAA Change	.400	0.132	0.152
Age	.500	0.157	0.187
Baseline HRSD_17_	.500	0.163	0.195
Treatment	.400	0.119	0.135
FAA Change *Treatment	.200	0.002	0.102

Note: Compares models that contain the effect to equivalent models stripped of the effect. Higher-order interactions are excluded.

**Table F6:** Bayesian Repeated Measures ANOVA for males, with factors and covariates Treatment Arm, Age and Baseline HRSD17.

Model comparison					
Models	P(M)	P(M|data)	BF_M_	BF_01_	error%
Null model (incl. subject)	.050	.189	4.416	1.000	
FAA Change	.050	.025	0.492	7.471	3.978
Treatment	.050	.303	8.262	0.622	0.600
FAA Change + Treatment	.050	.040	0.787	4.740	1.459
FAA Change + Treatment + FAA Change*Treatment	.050	.006	0.118	30.614	2.419
Age	.050	.047	0.929	4.045	1.842
FAA Change + Age	.050	.006	0.111	32.350	1.464
Treatment + Age	.050	.060	1.203	3.166	1.480
FAA Change + Treatment + Age	.050	.008	0.152	23.809	2.818
FAA Change + Treatment + Age + FAA Change*Treatment	.050	.001	0.022	162.929	2.264
Baseline HRSD_17_	.050	.081	1.684	2.316	2.516
FAA Change + Baseline HRSD_17_	.050	.010	0.201	18.048	1.736
Treatment + Baseline HRSD_17_	.050	.130	2.832	1.454	1.023
FAA Change + Treatment + Baseline HRSD_17_	.050	.018	0.339	10.743	2.659
FAA Change + Treatment + Baseline HRSD_17_ + FAA Change*Treatment	.050	.003	0.049	73.444	2.043
Age + Baseline HRSD_17_	.050	.028	0.547	6.740	1.240
FAA Change + Age + Baseline HRSD_17_	.050	.004	0.070	51.141	2.066
Treatment + Age + Baseline HRSD_17_	.050	.037	0.728	5.113	1.253
FAA Change + Treatment + Age + Baseline HRSD_17_	.050	.005	0.097	37.061	5.852
FAA Change + Treatment + Age + Baseline HRSD_17_ + FAA Change*Treatment	.050	7.334e-4	0.014	257.148	2.230

Note: All models include subject.

**Table F6:** Continued. Bayesian Repeated Measures ANOVA for males, with factors and covariates Treatment Arm, Age and Baseline HRSD17.

Analyses of effects			
Effects	P(incl)	P(incl|data)	BF_Inclusion_
FAA Change	.400	.116	0.132
Treatment	.400	.600	1.538
Age	.500	.195	0.243
Baseline HRSD_17_	.500	.316	0.462
FAA Change *Treatment	.200	.011	0.151

Note: Compares models that contain the effect to equivalent models stripped of the effect. Higher-order interactions are excluded.

**Fig G:**
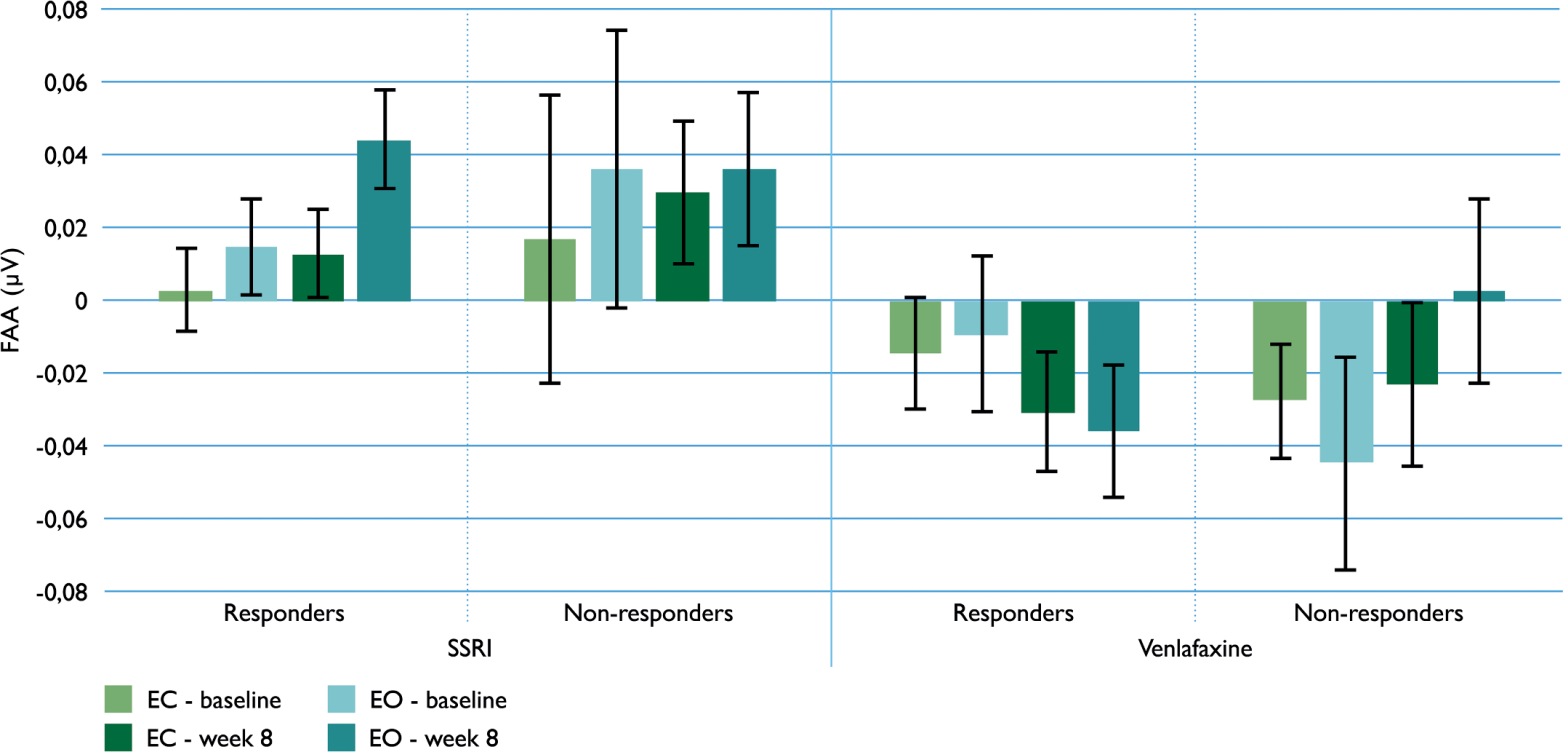
Mean values of male frontal alpha asymmetry (FAA, eyes open and eyes closed [EO and EC]), for the SSRI and venlafaxine groups, split up for responders and non-responders.

## Appendix A

Table A1.

## Appendix B. Comparison baseline and week-8 data

To justify the use of a follow-up sample that is supposed to contain the same MDD patients as the baseline data (paragraph 3.5), but does not due to incomplete assessments, we performed the baseline analysis from Arns et al. (2016) on only those who *did* have a complete week-8 assessment. The effect within the SSRI group was the same (*p* = .001, *F*(1,150) = 10.619, see Table B1 for all statistics).

## Appendix C

Table C1.

## Appendix D. VQIDS-SR_5_

To control for AD side effects, we repeated analyses from paragraph 3.2 and 3.3 and replaced all HRSD_17_ variables with VQIDS-SR_5_ equivalents. Correlational analyses showed that FAA Change was neither significantly correlated to the change score in VQIDS-SR_5_ (*r* = 0.059, *p* = .225), nor to the percentage change in VQIDS-SR_5_ (*r* = 0.060, *p* = .219).

Focusing on variables known to have an influence on FAA, specifically in the subgroup we thought to be prone to changes in FAA (severely depressed females and males over 53 years old), we did not find the FAA Change score to be significantly correlated to the change score in VQIDS-SR_5_, although subsample sizes were small. Extending the Repeated Measures model from paragraph 3.2 showed that VQIDS-SR_5_ baseline severity and age are not significantly contributing to FAA Change, both in males and females (see table D1 for all statistics).

## Appendix E

Table E1.

## Appendix F. Bayesian Repeated Measures ANOVA and correlations

### F1 Elaborated Bayesian analyses paragraph 3.2

Results of Bayesian testing of an absence of change in FAA, revealed a Bayes factor indicating evidence for the null hypothesis: the models with the factors FAA Change and Treatment Arm showed that the data occur >7.4 times more likely under the null hypothesis, than under any alternative model with (a combination of) the factors. This means that moderate evidence for the null hypothesis was found with only FAA Change in the model (BF_01_ = 7.483), increasing to (very) strong evidence when adding a combination of the two main effects (BF_01_ = 240.356) and including their interaction effect (BF_01_ = 5109.119). The error percentage was <2.5%, which indicates sufficient stability of the numerical algorithm that was used to obtain the result. For each factor, the BF_inclusion_ reflects how well the factor predicts the data by comparing the performance of all models that include the factor to the performance of all the models that do not include the factor. For both the factors FAA Change and Treatment Arm, there is weak evidence in favor of their inclusion (BF_inclusion_ = 0.134 and 0.031 respectively), as well as a weak evidence in favor of the inclusion of the interaction effect (BF_inclusion_ = 0.047). This implies that these factors are not providing evidence for change in FAA. See Table F1 for all results.

Bayesian Pearson correlations between FAA Change and the difference score HRSD_17_/the percentage difference HRSD_17_ reveal moderate to strong results, where the data are respectively 12.1 and 9.3 times more likely to occur under the null hypothesis than under the model assuming there is a correlation between the variables. See table F2 for all results.

### F2 Elaborated Bayesian analyses paragraph 3.3

Bayesian Repeated Measures ANOVAs for the two sex groups of severely depressed over the age of 53 reveal anecdotal (i.e. worth no more than a bare mention, a customary description for BFs ranging 1–3) to moderate results. Males: BF_01_ = 1.351–2.715 for models with only main effects, BF_01_ = 6.195 for the model with the interaction; BF_inclusion_ = 0.438–0.748; error% = 0.701–2.327. Females: BF_01_ = 1.864–2.944 for most models, BF_01_ = 4.304 for the model with only main effects of FAA Change and Treatment Arm; BF_inclusion_ = 0.434–1.462; error% = 0.922–1.372. Most models therefore provided no conclusive evidence for either the null or the alternative hypotheses, and BF_inclusion_s indicate that there is (very) weak evidence in favor of including the factors. However, some models indicated moderate evidence of the data being more likely to occur under the null hypothesis. See Tables F3 and F4 for all results.

Bayesian Repeated Measures alternatives for the extended ANOVAs showed similar results to paragraph 3.2: for females, the data are ≥6.6 times more likely to occur under the null hypothesis than under the alternative hypothesis (only models including factor FAA Change: BF_inclusion_ FAA Change and FAA Change X Treatment Arm 0.152 and 0.102, error % ≤ 8.576), and ≥4.7 times more likely in case of males (only models including factor FAA Change: BF_inclusion_ Time and Time X Treatment Arm 0.132 and 0.151, error% ≤ 5.582). See Tables F5 and F6 for all results.
